# Supplementary material for: Molecular and morphological evidence for a new species of Leptopus (Phyllanthaceae) from Southeast Yunnan, China
Source: PeerJ. 2021 Aug 24;9:e11989. doi: 10.7717/peerj.11989 (PMC8395577; doi:10.7717/peerj.11989)

华南农业大学植物标本馆
采 集 记 录

项目

采集号 YGYN202007150l 采集日期 2020年7月15日

采集人 姚纲

采集地 云南省麻栗坡县老山药王谷附近

经纬度 ， 海拔 约2000m

生境 路边、石坡、林下.

高(m) 性状

其他描述

[illegible]定

[illegible]料

[illegible]名

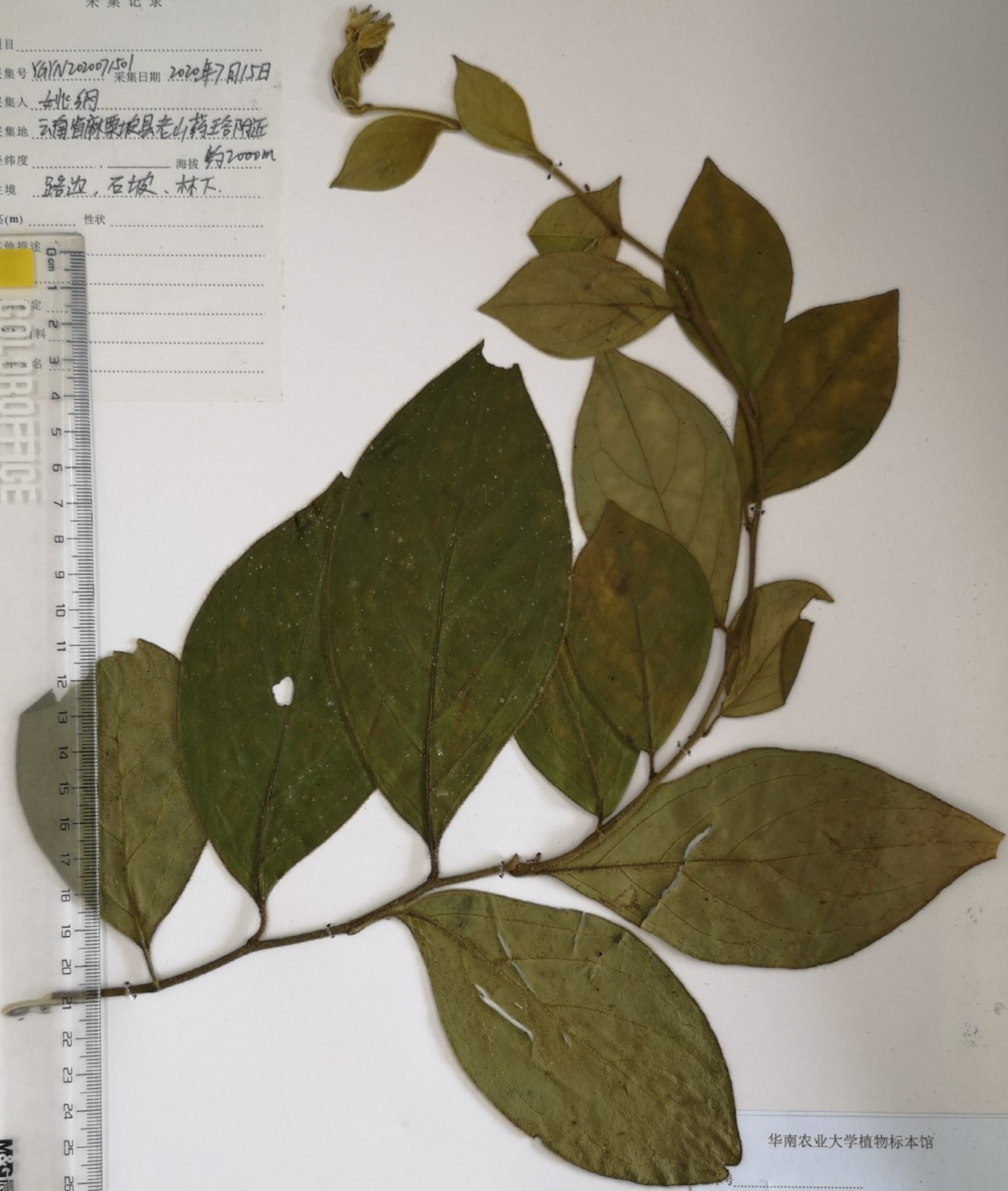

华南农业大学植物标本馆

[illegible]号

中文名 麻栗坡雀舌木

拉丁名 Leptopus malipoensis sp.

装订人 采集人 姚纲

鉴定人 姚纲 采集号 YGYN202007150l

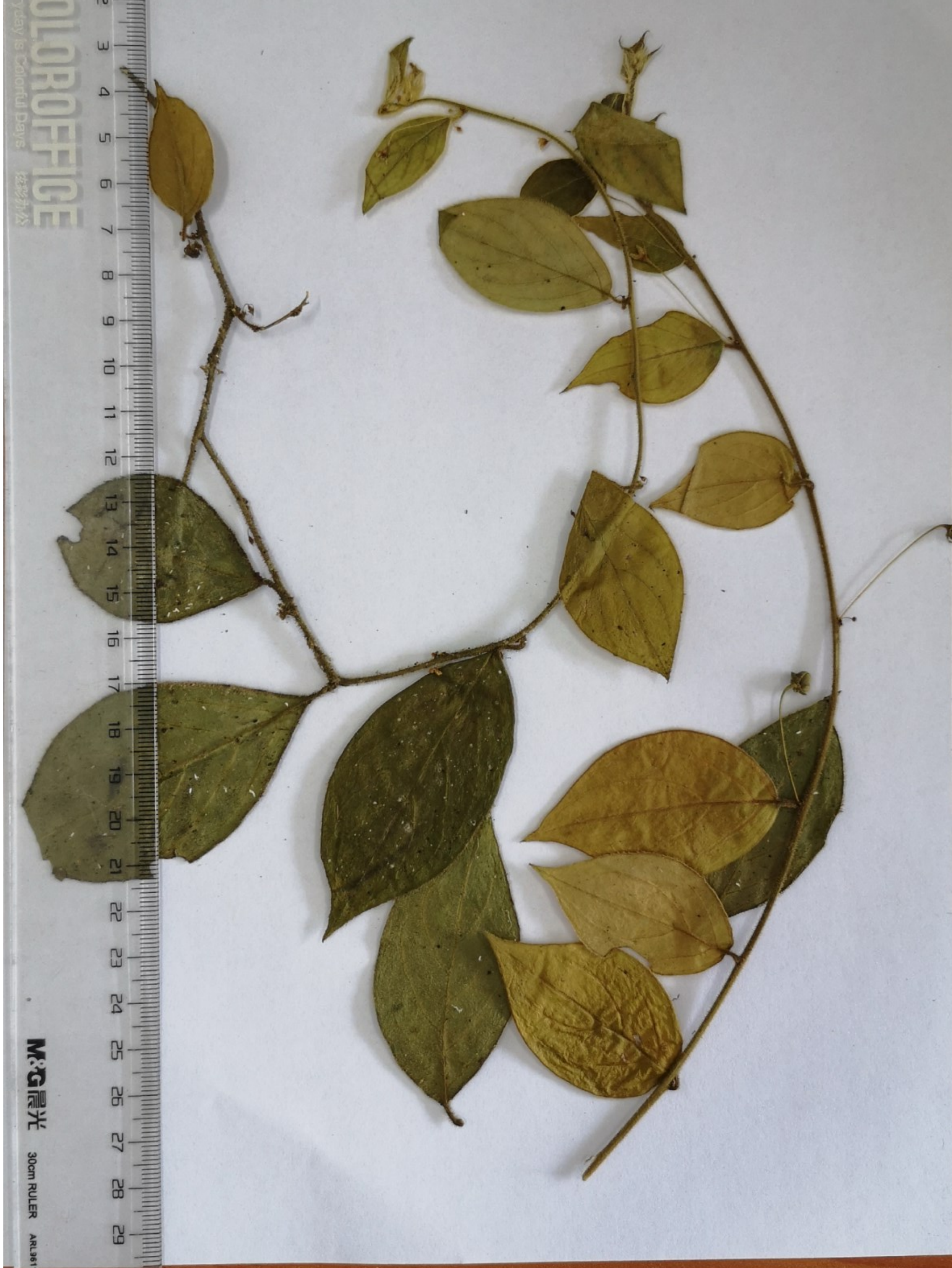
M&G晨光
30cm RULER

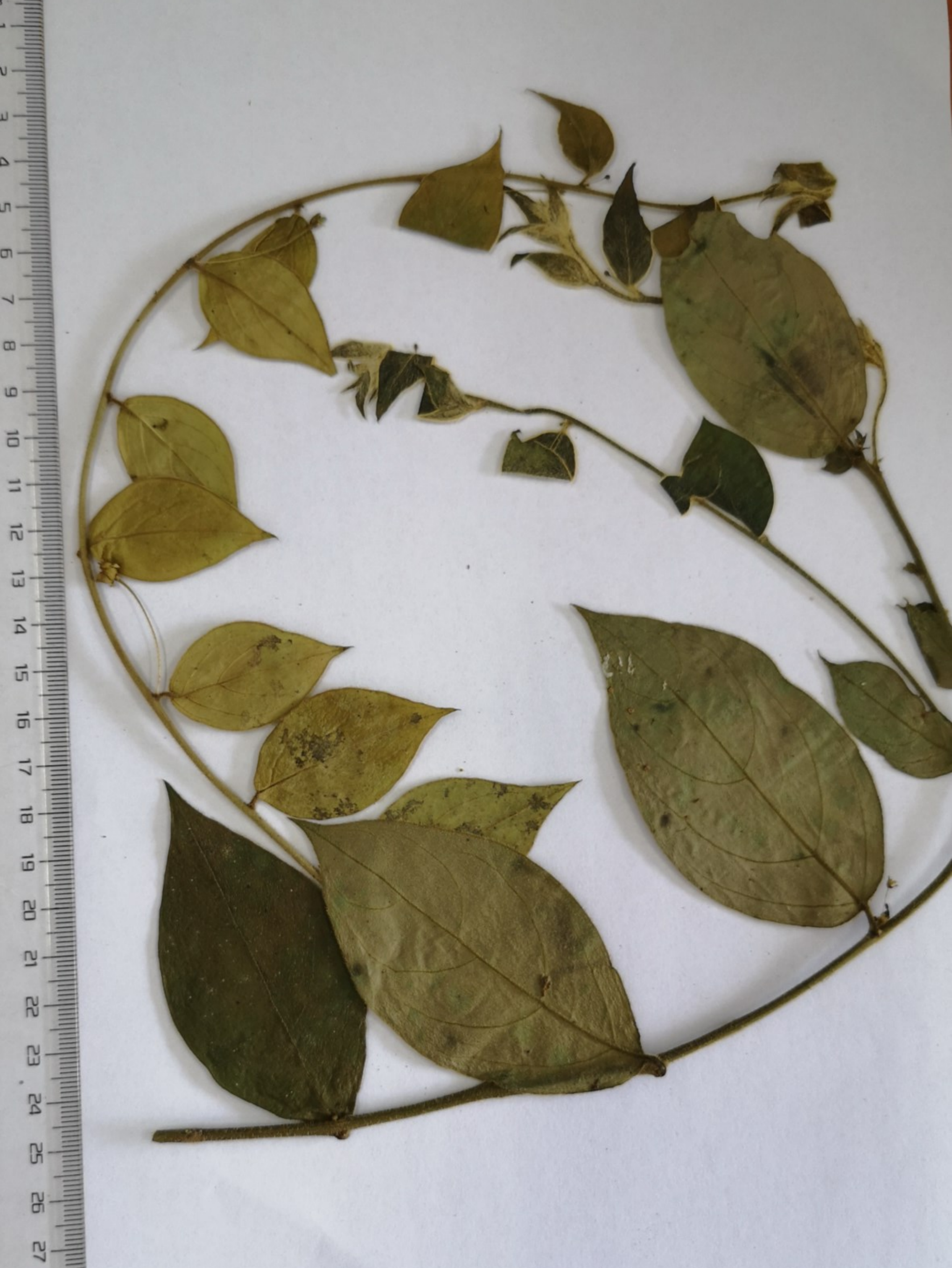

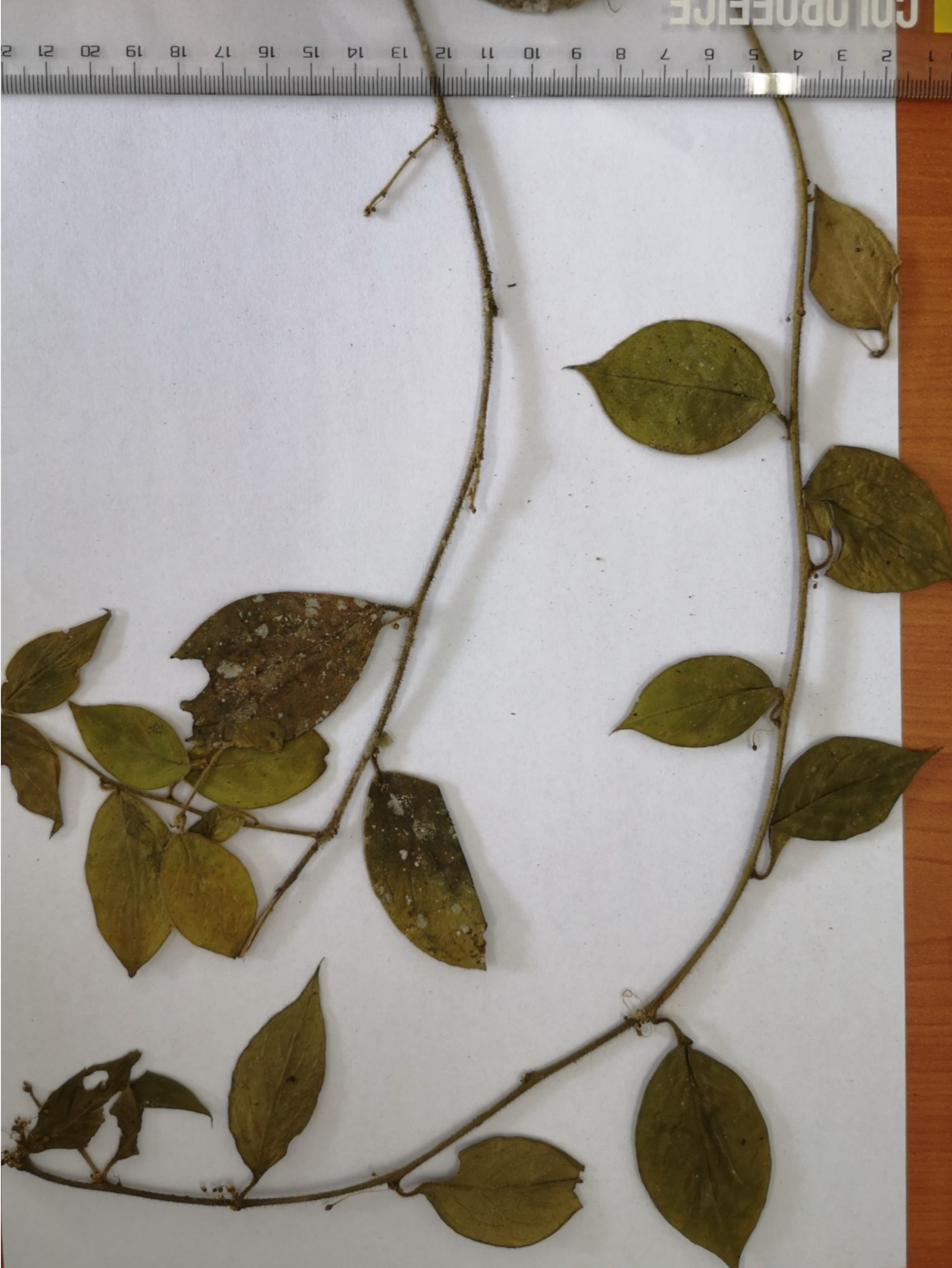

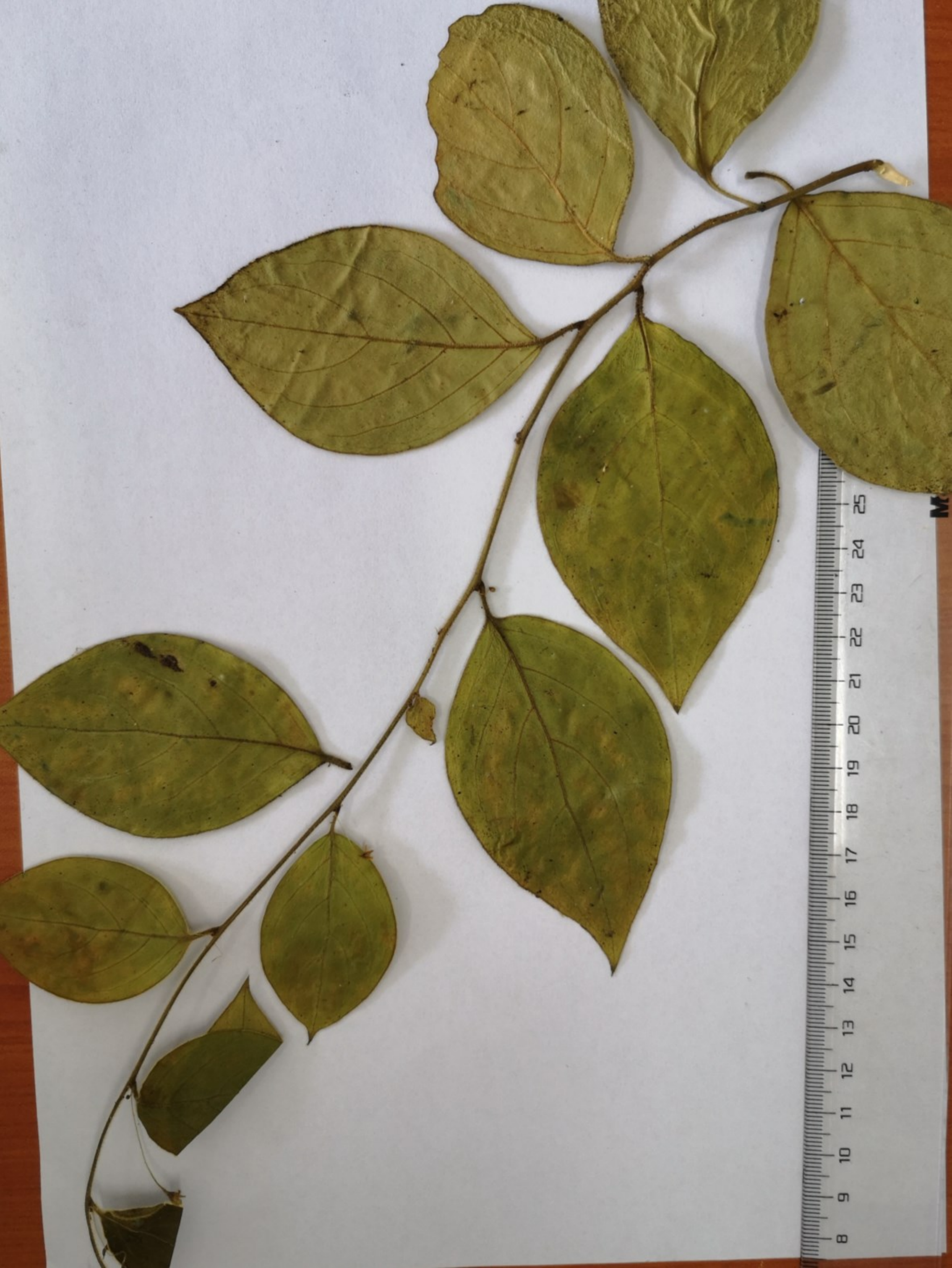
8
9
10
11
12
13
14
15
16
17
18
19
20
21
22
23
24
25

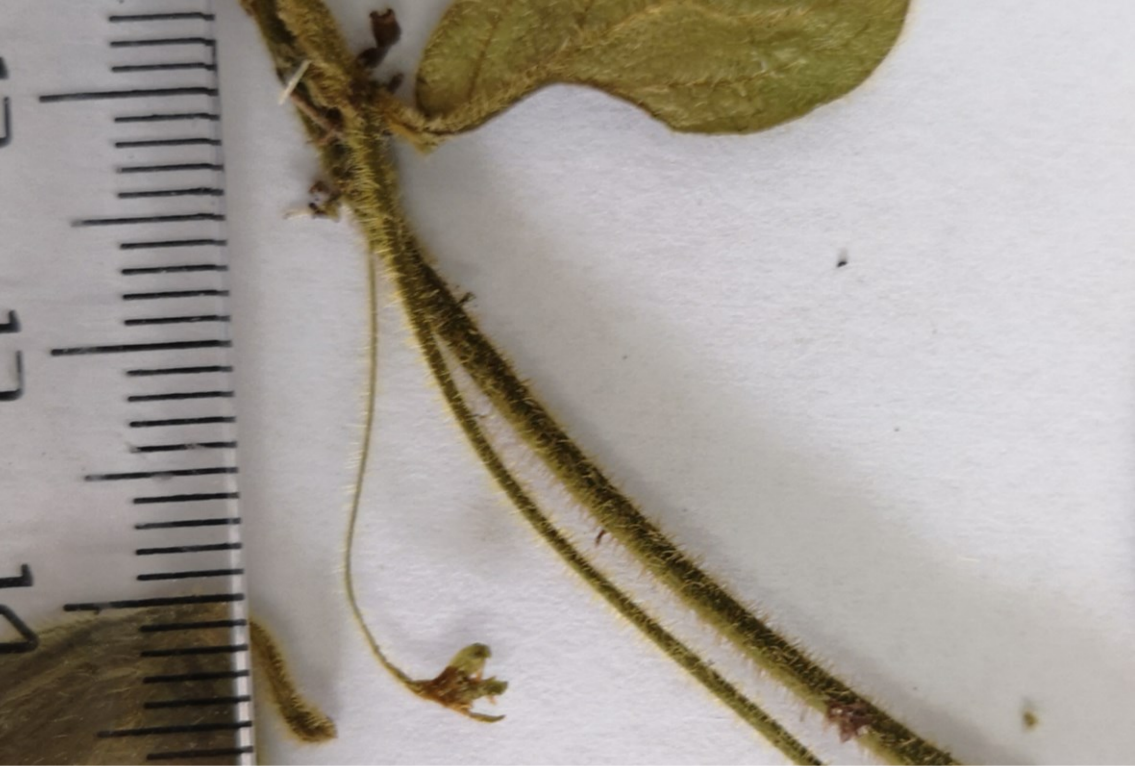

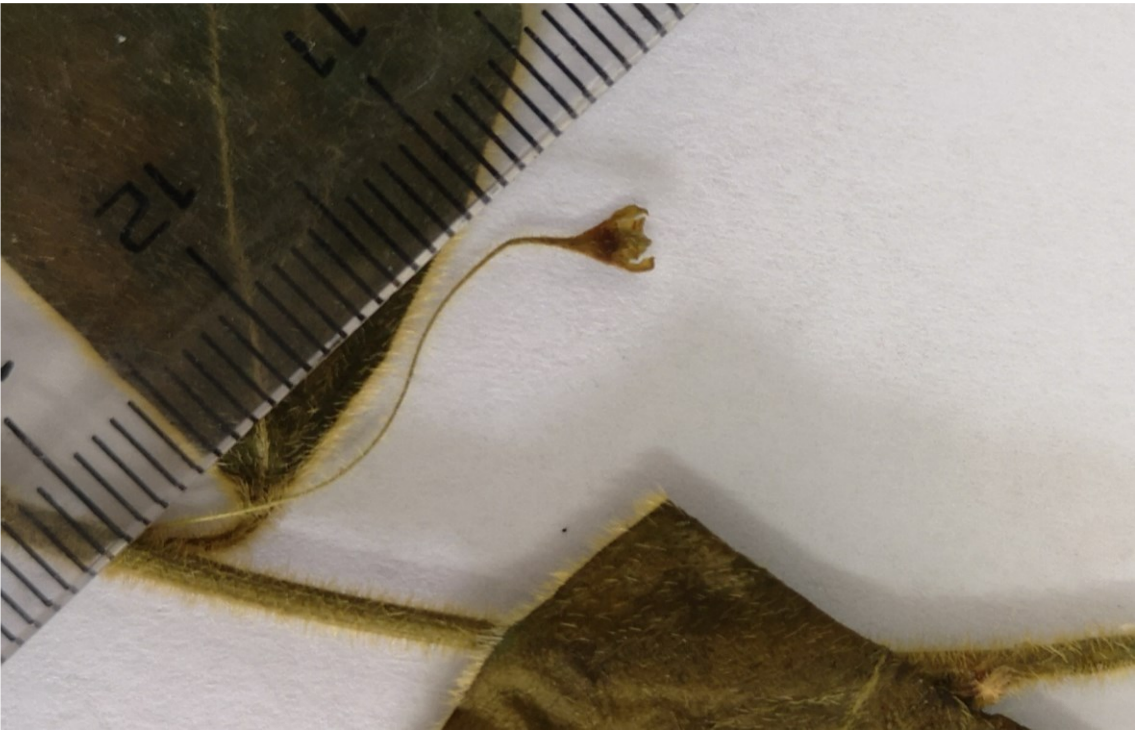

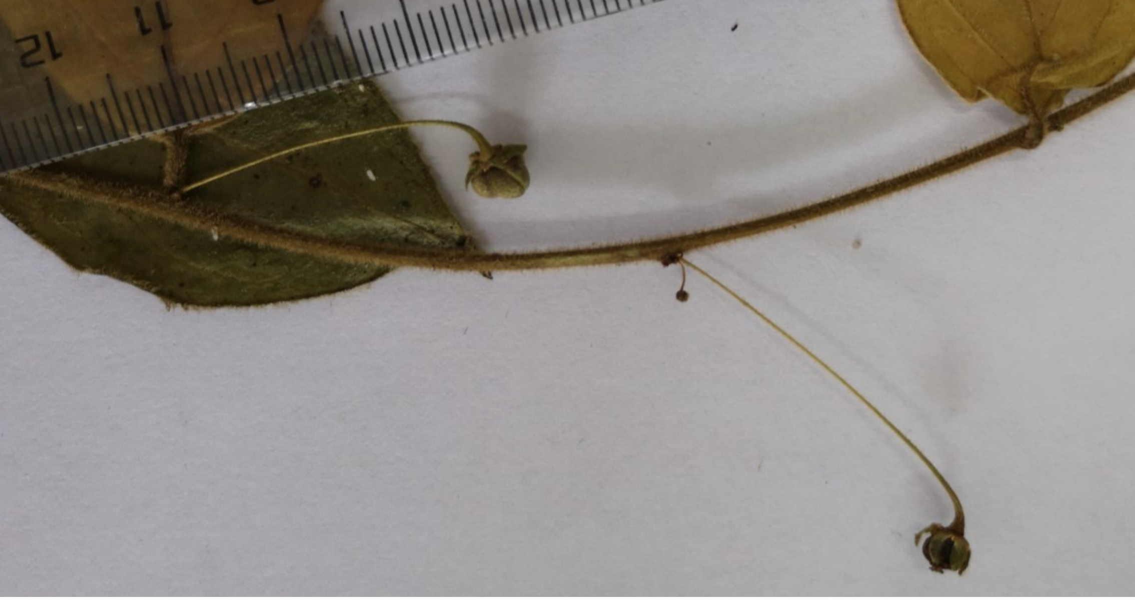

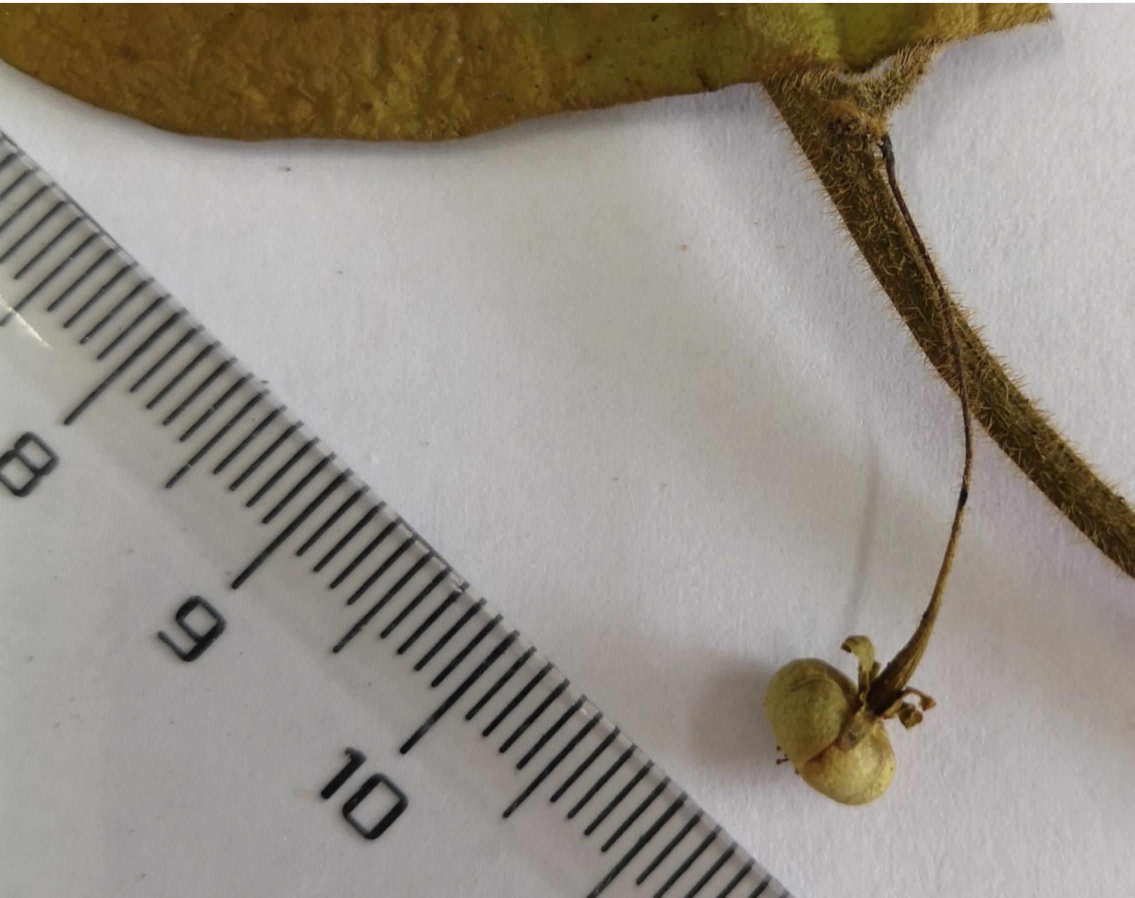

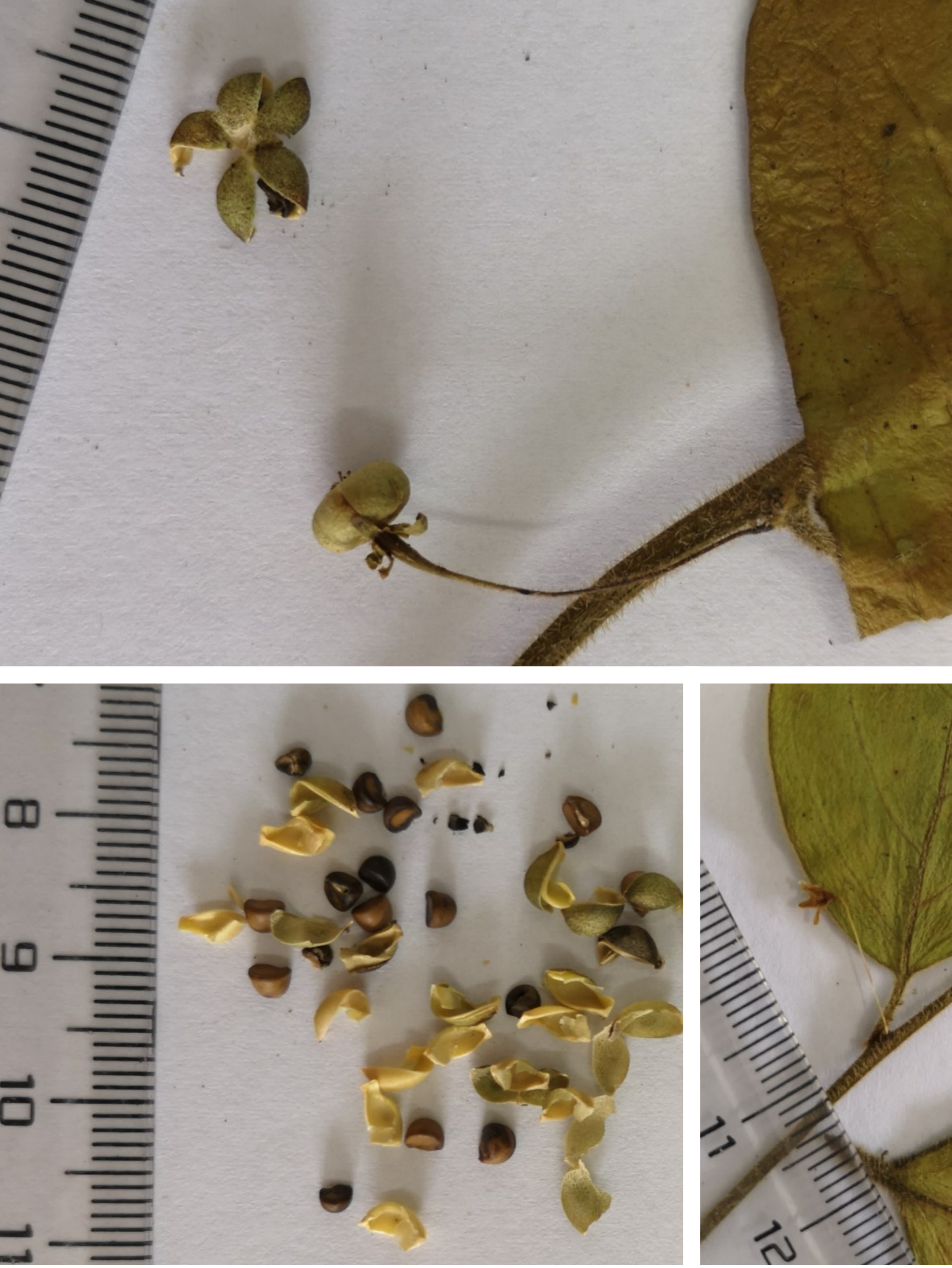

Supplement: Supplemental Information 6 [file peerj-09-11989-s006.pdf]
